# Early Prediction of Unplanned 30-Day Hospital Readmission: Model Development and Retrospective Data Analysis

**DOI:** 10.2196/16306

**Published:** 2021-03-23

**Authors:** Peng Zhao, Illhoi Yoo, Syed H Naqvi

**Affiliations:** 1 Institute for Data Science and Informatics University of Missouri Columbia, MO United States; 2 Department of Health Management and Informatics School of Medicine University of Missouri Columbia, MO United States; 3 Division of Hospital Medicine Department of Medicine University of Missouri School of Medicine Columbia, MO United States

**Keywords:** patient readmission, risk factors, unplanned, early detection, all-cause, predictive model, 30-day, machine learning

## Abstract

**Background:**

Existing readmission reduction solutions tend to focus on complementing inpatient care with enhanced care transition and postdischarge interventions. These solutions are initiated near or after discharge, when clinicians’ impact on inpatient care is ending. Preventive intervention during hospitalization is an underexplored area that holds potential for reducing readmission risk. However, it is challenging to predict readmission risk at the early stage of hospitalization because few data are available.

**Objective:**

The objective of this study was to build an early prediction model of unplanned 30-day hospital readmission using a large and diverse sample. We were also interested in identifying novel readmission risk factors and protective factors.

**Methods:**

We extracted the medical records of 96,550 patients in 205 participating Cerner client hospitals across four US census regions in 2016 from the Health Facts database. The model was built with index admission data that can become available within 24 hours and data from previous encounters up to 1 year before the index admission. The candidate models were evaluated for performance, timeliness, and generalizability. Multivariate logistic regression analysis was used to identify readmission risk factors and protective factors.

**Results:**

We developed six candidate readmission models with different machine learning algorithms. The best performing model of extreme gradient boosting (XGBoost) achieved an area under the receiver operating characteristic curve of 0.753 on the development data set and 0.742 on the validation data set. By multivariate logistic regression analysis, we identified 14 risk factors and 2 protective factors of readmission that have never been reported.

**Conclusions:**

The performance of our model is better than that of the most widely used models in US health care settings. This model can help clinicians identify readmission risk at the early stage of hospitalization so that they can pay extra attention during the care process of high-risk patients. The 14 novel risk factors and 2 novel protective factors can aid understanding of the factors associated with readmission.

## Introduction

Unplanned hospital readmission continues to attract much attention due to its negative influence on patients’ quality of life and substantial contribution to health care costs. During July 2015 to June 2016, 15.2% of Medicare beneficiaries experienced unplanned readmission within 30 days after discharge [1]. It has been estimated that unplanned readmission accounts for US $17.4 billion in Medicare expenditures annually [2]. In an effort to improve health care quality and decrease unplanned hospital readmission rates, the Affordable Care Act [3] implemented the Hospital Readmission Reduction Program (HRRP) [4] in 2012 to use unplanned 30-day hospital readmission as a metric to financially penalize hospitals with excessive readmission rates. The high associated cost and penalties from the HRRP have intensified the efforts of the entire health care industry to reduce unplanned hospital readmissions.

Existing readmission reduction interventions, especially transition interventions and postdischarge interventions, focus on complementing inpatient care with enhanced services; however, the planning, implementation, and monitoring of these interventions can be resource-intensive [5]. In addition, no single intervention or bundle of interventions were found to be reliable in reducing readmissions, according to the review by Hansen et al [6]. Another disadvantage is that these interventions do not greatly impact the quality improvement of inpatient care because they are mostly initiated near or after discharge, when clinicians’ impact on inpatient care is ending. Preventive intervention during hospitalization is an underexplored area that holds potential for reducing readmission risk. It has been shown that early interventions during inpatient hospitalization, such as early discharge planning [7], can reduce readmissions. However, it is impractical to deliver readmission-preventive interventions to all patients because health care resources are restricted. Predictive modeling is an efficient method to optimize the allocation of valuable clinical resources by stratifying patients’ readmission risk and targeting the delivery of preventive interventions to patients at high risk [8]. Evidence has shown that focusing interventions on high-risk patients can reduce 30-day hospital readmission risk by 11%-28% [9-11]. However, the majority of reported hospital readmission predictive models have limited value in real-world health care settings because they require variables whose values only become completely available at discharge [12]. For example, the HOSPITAL score [13] and the LACE index [14] are the most widely used readmission risk calculators in US healthcare settings. They only work at the end of inpatient care because they require variables that are not available in a timely fashion, such as the length of stay and the results of some laboratory tests before discharge. It is essential to perform early risk assessments of high-risk patients to enable clinicians to deliver timely preventive interventions at the early stage of hospitalization [15].

Several 30-day hospital readmission early detection models have been reported; however, their performance and design are unsatisfactory. Wang et al [16] developed a real-time readmission model using a time series of vital signs and discrete features such as laboratory tests. However, this model was a black box, and it was unclear how the clinical factors led to the predictions. In health care applications, the interpretability of a model is as important as its performance because the attributes and the decision path must be medically rational. Horne et al [17] developed a laboratory-based model specific to heart failure patients. It can be used within 24 hours of admission; however, the performance was poor, with areas under the receiver operating characteristic curve (AUCs) [18] of 0.571 and 0.596 in female and male validation data sets. Cronin et al [19] reported an early detection model based on the information available at admission and medications used in index admission; it showed a moderate performance in the validation data set, with an AUC of 0.671. El Morr et al [20] created a modified LACE index (LACE-rt) to support real-time prediction by replacing the length of stay during the current admission in the original LACE index with that of the previous admission within the last 30 days. However, this model only showed fair performance (AUC 0.632) [20]. Shadmi et al [21] developed an early prediction model for emergency readmissions based on data available before the index admission, and they achieved an AUC of 0.69 in the validation data set. The same team further modified the model by adding risk factors accrued during index admissions; however, they obtained a similarly moderate AUC (0.68) in the validation data set [22]. Amarasingham *et al* [23] reported a real-time readmission model (AUC of 0.69 in the validation data set) for patients with heart failure using clinical and social factors available within 24 hours of admission. However, their cohort size was too small, with only 1372 index admissions.

The objective of this work was to build a predictive model for early detection of unplanned 30-day hospital readmission using a large and diverse sample. We were also interested in identifying novel risk factors and protective factors of readmission. We used machine learning methods to develop a predictive model that can monitor readmission risk at the early stage of hospitalization. Unlike most models, which focus only on characteristics of index admissions, we included the detailed medical history of previous encounters up to 1 year before index admissions to construct a better readmission prediction model.

## Methods

### Study Design

**Figure 1:**
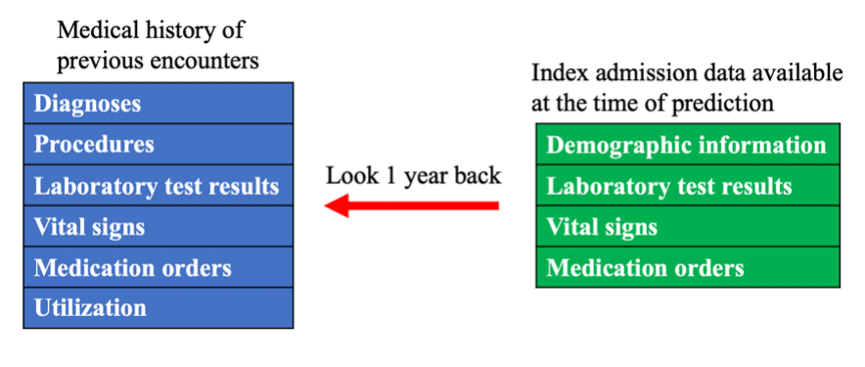
The variables used to develop the models.

This study was a retrospective analysis of electronic health record (EHR) data. To ensure the readmission prediction model can be accurate at the early stage of hospitalization, we only used index admission attributes whose values can become available in the EHR within 24 hours, including patient demographics, laboratory test results, vital signs, and medication orders. The patients’ data were enriched by the detailed history of previous hospital encounters within one year before the current inpatient stay, including the information of diagnoses, procedures, laboratory test results, vital signs, medication orders, and health care utilization. Figure 1 shows the types of variables used for modeling.

### Data Source

The data were extracted from Health Facts [24], an EHR database curated by Cerner Corporation. This Health Insurance Portability and Accountability Act (HIPAA)–compliant database was collected from participating Cerner client hospitals and from clinics with de-identified longitudinal records of diagnoses, laboratory test results, surgeries, microbiology test results, medications, medical events, and medical histories. The version accessed by researchers at the University of Missouri contained 3.15 TB of encounter-level medical records extracted from 782 hospitals and clinics in the United States between 2000 and 2016.

### Ethics

This retrospective data analysis study was not required to obtain approval from the University of Missouri Institutional Review Board because the data used in the study were already fully deidentified by the data owner (Cerner Corporation).

### Data Inclusion and Exclusion Criteria

The data inclusion and exclusion criteria were based on the criteria used by the Centers for Medicare & Medicaid Services (CMS) [25] with minor modifications. (1) We captured inpatient encounters between January 1 and December 31, 2016, in acute care hospitals with a length of stay longer than 24 hours. (2) The gap between index admission discharge and readmission was between 1 and 30 days (inclusive). (3) If a patient had more than one inpatient visit within 30 days of discharge, only the first visit was considered as readmission. (4) Patients were aged older than 18 years at admission. (5) Patients were not transferred to other acute care facilities and were alive at discharge. (6) Patients were not readmitted for newborn status, labor, accident, trauma, rehabilitation services, or other scheduled care according to the CMS planned readmission identification algorithm [25]. (7) In this work, we adopted the concept of “hospital-wide all-cause readmission” used by CMS [26] to study readmissions due to medical and health care–related reasons. (8) Patients without readmissions had the same requirements for their index admissions. (9) Each patient had only one record.

### Feature Engineering and Data Transformation

According to our previous literature review of readmission risk factors [27], patients’ demographic and social factors as well as their previous health care utilization were strong predictors for readmission. In this work, we incorporated the patients’ age at admission, sex, race, insurance payer, hospital census region, census division, rurality, and health care utilization in the previous year, including the number of inpatient visits, outpatient visits, emergency department visits, and times the patient left the hospital against medical advice. We only retained records without any missing demographic information. We also investigated the impact of the patients’ medical history within the year before the index admission. We used counts to condense longitudinal medical histories into structured data so that patients with different medical histories could be represented in the same feature space. Patients with a medical history had higher counts, and patients without any medical history had counts of zero. In this way, we were able to handle the missing value problem for new patients. For example, if a patient had the same diagnosis of heart failure in two separate encounters in the previous year, this diagnosis would have a count of two. For laboratory tests and vital signs, the latest results were checked and recorded if they were abnormal. Suppose a patient’s systolic blood pressure was taken twice in one encounter, and the result of the second test was abnormal. In another encounter, it was taken three times, and the latest result was normal. This patient would be determined to have had one abnormal systolic blood pressure result during the two encounters in the last year. For the index admission, we only checked the medication record and the latest results of laboratory tests and vital signs. Diagnosis codes were mapped from the *International Classification of Disease, Tenth Revision, Clinical Modification* (*ICD-10-CM*) [28] into the Clinical Classifications Software (CCS) categories [29] because the ICD codes were too granular for data mining purposes. For the same reason, procedure codes were mapped from the *International Classification of Disease, Tenth Revision, Procedure Coding System* (*ICD-10-PCS*) [28], current procedural terminology (CPT) [30], and Healthcare Common Procedure Coding System (HCPCS) [31] codes into CCS categories. Laboratory tests and vital signs were represented by their original names. We used generic names to represent medication orders. Table 1 shows an explanation of these features. After data transformation and feature engineering, the final data set contained 432 variables.

**Table 1:** Feature representation and value types.

Type and category			Representation		Data type
**Medical history in last year**					
	Diagnosis	CCS^a^		Count	
	Procedure	CCS		Count	
	Laboratory test	Name		Count	
	Vital sign	Name		Count	
	Medication	Generic name		Count	
	Utilization	Name		Count	
**Index admission**					
	Demographic	Name		Discretized age, race, sex, payer, region, or rurality	
	Medication	Generic name		Ordered or not	
	Laboratory test	Name		Latest result is abnormal or not	
	Vital sign	Name		Latest result is abnormal or not	

^a^CCS: Clinical Classifications Software.

### Candidate Algorithms and Baseline Models

Interpretability is an important consideration for clinical predictive models because it is crucial to ensure medical rationality in the classification process. We selected six candidate machine learning algorithms that can generate probabilistic outputs, including logistic regression, naïve Bayes, decision tree, random forest, gradient boosting tree, and artificial neural networks. Logistic regression belongs to the family of generalized linear models [32], and it predicts the log odds of the positive class as a linear combination of variables weighted by coefficients [33]. The association of a variable (factor) with the response target can be measured by the odds ratio [34], which is equal to the exponential of the coefficient of the variable. An odds ratio >1 indicates that the presence of the factor increases the odds of the outcome (eg, readmission). Naïve Bayes is a probabilistic classification algorithm based on the Bayes theorem [35] with the assumption that variables are independent [36]. Classifications are achieved by assigning the class label that can maximize the posterior probability given the features of an instance. A naïve Bayes model can be interpreted by taking the conditional probability of a variable given a class, and a higher probability indicates a stronger relationship with the class. Decision trees are a family of tree-structured predictive algorithms that iteratively split the data into disjoint subsets in a greedy manner [37]. Classifications are made by walking the tree splits until arriving at a leaf node (the class). Decision trees are self-explainable because each leaf node is represented as an if-then rule, and the decision process can be visualized. The contribution of a variable to the classification can be measured using various methods, such as information gain based on information theory and Gini importance [38]. Random forests are ensemble learning algorithms generated by bootstrap aggregation; the algorithm repeatedly selects a random sample from the training data set (with replacement) and builds a decision tree for the sample [39]. When making predictions, the outputs from different decision trees will be ensembled. Gradient boosting trees are another type of tree ensemble algorithm; they build the model in a stagewise fashion by iteratively generating new trees to improve the previous weaker trees [40]. Predictions are made by the weighted average of tree outcomes, with stronger trees having higher weights. Random forests and gradient tree boosting algorithms can be interpreted by measuring the Gini importance of the variables. Artificial neural networks are an interconnected group of computing units called artificial neurons [41]. The artificial neurons are aggregated into layers and connected by edges that have different weights to control the signals transmitted between neurons. The signals in the final output layer are used for prediction. The importance of each feature can be measured by the increase in prediction error after permuting the values of the feature.

We implemented the HOSPITAL score, LACE index, and LACE-rt index to compare their performance with that of our models. The HOSPITAL score has seven variables, including hemoglobin level at discharge, discharge from an oncology service, sodium level at discharge, any ICD procedures during the hospital stay, the type of index admission, the number of admissions 1 year before the index admission, and the length of stay [13]. Each factor level has a weighted point, and the maximum total score is 13 points. The LACE index has four variables, including length of stay, acuity of admission, the Charlson comorbidity index, and the number of emergency department visits 6 months before the index admission [14]. Its score ranges from 0 to 19 points. The LACE-rt index has the same variable weights and the same maximum score as the original LACE index [20]. The only difference is that it requires the length of stay during the previous admission within last 30 days instead of the current admission.

### Model Training and Benchmark

Based on the inclusion and exclusion criteria, we identified 96,550 eligible patients. We randomly split the 96,550 records into a development data set (91,550 records) and a validation data set (5000 records). The readmission rate (11.7%) was preserved in these two data sets. The development set was used to derive and test the five candidate models in 10-fold cross-validation. The validation set was used to assess if the models can be generalized to unseen data. For the three baseline models, we extracted the required variables from the encounters in the validation set so that we could perform a fair comparison of our candidate models and the baseline models.

Because accuracy is sensitive to class imbalance, it cannot be used to evaluate readmission models (readmission rate <50%). To measure the performance of the models, we used the AUC, precision, recall, specificity, and F1 measure, which are less sensitive to data imbalance. The AUC is the probability that a model will rank a randomly chosen positive instance higher than a randomly chosen negative instance. The AUC ranges from 0.5 to 1.0, with 1.0 indicating that the model has perfect discrimination ability and 0.5 indicating that it performs no better than random guessing. Precision is the fraction of true positives among all instances predicted to be positive. Recall is the fraction of correctly identified positives in all positive instances. Specificity is the fraction of correctly identified negatives in all negative instances. The F1 measure is the harmonic mean of precision and recall. The values of the precision, recall, specificity, and F1 measure range from 0 to 1.0. A higher value indicates better performance.

### Multivariate Logistic Regression Analysis

We performed multivariate logistic regression analysis [32] to evaluate associations between the independent variables and the patients’ readmission status. Features were selected by backward elimination. We chose the significance level of .05 for the statistical tests.

### Software

We used Weka [42] to build and evaluate the logistic regression, naïve Bayes, decision tree, random forest, and gradient boosting tree models. The extreme gradient boosting (XGBoost) model and neural network models were developed in Python. The hyperparameters of these models were optimized. We implemented the HOSPITAL score, LACE index, and LACE-rt index in Python. The multivariate logistic regression analysis used the GLM package in R (R Project).

## Results

### Patient Demographics

From the 96,550 included patients, 11,294 experienced unplanned 30-day hospital readmission. The readmission rate (11.7%) is lower than the Medicare readmission rate (15.2% [1]). One possible reason was that in contrast to Medicare patients, who are normally older than 65 years, our study population included younger and less vulnerable adult patients (aged 18 to 64 years) as well as older adults (aged 65 years and above). Table 2 shows the demographic information of patients with and without readmissions. Most patients were White, female, and between 65 and 79 years of age.

**Table 2:** Demographic information of the 96,550 patients included in the data set. The characteristics with the highest frequencies are indicated with italic text.

Characteristic			Value, n (%)		
		Readmission=yes		Readmission=no	
Total			11,294 (11.7)		85,256 (88.3)
**Age (years)**					
	18-34	930 (8.2)		13,242 (15.5)	
	35-49	1525 (13.5)		12,541 (14.7)	
	50-64	3116 (27.6)		21,559 (25.3)	
	65-79*	*3380 (29.9)*		*22,634 (26.5)*	
	≥80	2343 (20.7)		15,280 (17.9)	
**Sex**					
	*Female*	*5966 (52.8)*		*49,619 (58.2)*	
	Male	5328 (47.2)		35,637 (41.8)	
**Race**					
	African American	2612 (23.1)		16,248 (19.1)	
	*White*	*7750 (68.6)*		*61,685 (72.3)*	
	Other	932 (8.3)		7323 (8.6)	

*Italicized text represents majority in the group.

### Model Development and Selection

Table 3 shows the 10-fold cross-validation AUCs (mean and standard deviation) of the six candidate models on the development data set. Especially, the alternating decision tree (ADTree) algorithm [43], the XGBoost [44] algorithm, and the feedforward neural networks with three hidden layers (256 neurons, 512 neurons, and 256 neurons) had the best AUCs within the decision tree, gradient boosting tree, and artificial neural network families, respectively.

**Table 3:** 10-fold cross-validation AUCs of the candidate models on the development set.

Model	10-fold cross-validation AUC^a^, mean (SD)
Logistic regression	0.750 (0.005)
Naïve Bayes	0.730 (0.006)
Alternating decision tree	0.730 (0.010)
Random forest	0.734 (0.006)
XGBoost^b^	0.753 (0.007)
Neural network	0.746 (0.004)

^a^AUC: area under the receiver operating characteristic curve.

^b^XGBoost: extreme gradient boosting.

We further compared the performance of the six candidate models and the three baseline models (HOSPITAL score, LACE index, and LACE-rt index) on the validation data set by measuring the precision, recall, specificity, F1 measure, and AUC (Table 4). Because of the imbalanced prevalence of readmissions (eg, 11.7% in this study), it was infeasible to use 0.5 as the cutoff probability to dichotomize probabilistic outputs. We chose cutoffs that could maximize the Youden index of each model [45]. The optimal cutoffs of the three baseline models are integers because they do not generate probabilities. It can be seen that the random forest model has the best specificity and precision, while the XGBoost model has the best recall, F1 measure, and AUC. In the medical domain, recall is a more important metric because false negatives are considered more risky than false positives. Therefore, although the XGBoost and logistic regression models had similar AUC on development set (Table 3) and validation set, we chose XGBoost as the final model. It can be seen that the XGBoost model is better than the three baseline models in all performance metrics. Features of the XGBoost model and their importance ranking are shown in Multimedia Appendix 1. The optimized XGBoost model has “gbtree” as the booster, “binary:logistic” as the objective, a gamma of 0.4, a learning rate of 0.1, a maximum depth of 3, a maximum delta step of 0, a minimum child weight of 8, a reg_alpha parameter of 5, a reg_lambda parameter of 1, a subsample of 0.7, and 240 estimators.

**Table 4:** Performance of the candidate models and baseline models on the validation set. The best-performing parameters are indicated in italic text.

Model	Optimal cutoff	Specificity	Precision	Recall	F1 measure	AUC^a^
Logistic regression	0.157	0.642	0.857	0.729	0.773	0.741
Naïve Bayes	0.220	0.666	0.855	0.685	0.740	0.720
Alternating decision tree	0.298	0.662	0.857	0.705	0.755	0.732
Random forest	0.122	*0.747*	*0.862*	0.611	0.680	0.726
XGBoost^b^	0.175	0.611	0.856	*0.759*	*0.794*	*0.743*
Neural network	0.125	0.686	0.858	0.681	0.737	0.735
HOSPITAL score	4	0.564	0.838	0.694	0.745	0.688
LACE index	11	0.469	0.830	0.745	0.779	0.675
LACE-rt index	7	0.542	0.833	0.688	0.740	0.668

^a^AUC: area under the curve.

^b^XGBoost: extreme gradient boosting.

### Risk Factors and Protective Factors of Readmission

To understand the statistical significance of the factors, we performed multivariate logistic regression analysis on all the data (96,550 records and 432 variables). By backward elimination, we reduced the feature space to 83, as shown in Multimedia Appendix 2. We reidentified 40 risk factors and significant predictors reported in previous studies. In addition, we discovered 14 risk factors and 2 protective factors that have never been reported in the literature. These 16 novel factors belong to 13 variables, and they are displayed in Table 5.

**Table 5:** The 14 novel risk factors and 2 novel protective factors of readmission identified in the study.

Risks or protective factors				Coefficient		*P* value		Odds ratio (95% CI)
**Medical history in last year**								
	**Diagnosis**							
		1 maintenance chemotherapy visit in the last year	0.390			<.001	1.476 (1.218-1.790)	
	**Laboratory test result**							
		1 abnormal lymphocyte count test in the last year			0.221	<.001		1.247 (1.144-1.359)
		≥2 abnormal lymphocyte count tests in the last year			0.228	.001		1.257 (1.091-1.447)
		1 abnormal monocyte count test in the last year	0.182			.005	1.199 (1.056-1.362)	
		≥2 abnormal monocyte percent tests in the last year	0.316			<.001	1.371 (1.178- 1.596	
		1 abnormal serum calcium quantitative test in the last year			0.226	<.001		1.254 (1.107-1.420)
		≥2 abnormal serum calcium quantitative tests in the last year			0.297	.001		1.345 (1.122-1.612)
	**Medication**							
		1 albuterol ipratropium order in the last year			0.071	.02		1.073 (1.010-1.141)
		≥2 albuterol ipratropium orders in the last year			0.145	.003		1.157 (1.052-1.272)
		1 cefazolin order in the last year			–0.123	.001		0.884 (0.822-0.950)
**Index admission**								
	**Demographic information**							
		Index admission to hospital in Northeast census region			0.365	<.001		1.441 (1.345-1.543)
	**Medication**							
		Gabapentin ordered at index admission			0.162	<.001		1.176 (1.113-1.243)
		Ondansetron ordered at index admission			0.105	<.001		1.111 (1.057-1.168)
		Polyethylene glycol 3350 ordered at index admission			0.073	.01		1.076 (1.017-1.139)
		Cefazolin ordered at index admission			–0.147	<.001		0.863 (0.798-0.934)
	**Laboratory test result**							
		≥16 abnormal laboratory test results at index admission			0.140	.005		1.151 (1.043-1.269)

## Discussion

### Novel Risk Factors and Protective Factors of Readmission

The 14 novel risk factors and 2 novel protective factors of readmission are related to medical history and index admission. They belong to four categories: diagnosis, laboratory test results, medications, and demographic information.

Patients with one CCS-level diagnosis of maintenance chemotherapy in the previous year were found to be more likely to be readmitted than patients without this diagnosis. This can be explained by the linkage between chemotherapy and cancer, which has been reported as a predictor of readmission [46,47].

A blood disorder or an abnormal amount of substance in the blood can indicate certain diseases or side effects. Having an increased number of abnormal test results indicates that the patient is frailer and can be more prone to readmission.

Four medications were found to be positively linked to readmission. These medications may have side effects that are associated with readmission. Another interpretation is that conditions treated by these medications may be related to readmission. For example, albuterol ipratropium is a combination of two bronchodilators, which are used in the treatment of chronic obstructive pulmonary disease (COPD). COPD has been reported as a risk factor of readmission [47]. It is interesting that the prescriptions of cefazolin in previous encounters and at index admission were both negatively associated with readmission. One possible explanation is that cefazolin is an antibiotic that is used to treat infections caused by bacteria. The use of cefazolin may reduce patients’ chance of infection and reduce their readmission risk.

The Northeast census region was found to be more positively associated with readmission than the Midwest census region. One possible reason is that geolocation is associated with socioeconomic status, which has been reported to be linked to readmission [48].

### Timeliness of Prediction

Most readmission predictive models are based on index admission data. Many highly predictive variables of the index admission, such as the length of stay, diagnosis codes, procedure codes, and laboratory test results before discharge, are only available near or after discharge. To achieve good predictive performance, most studies include these variables in their models. As a result, these models can only be used near or after discharge. They are useful for public reporting but not for clinical decision support because they are not timely.

In this work, we used the data from index admission and patients’ medical history up to 1 year before the index admission. To ensure that the model could work at any time during hospitalization, we only used index admissions data that become available in the EHR within 24 hours during hospitalization, such as medication orders and laboratory test results. We used the detailed medical history from the patients’ previous encounters. Although some studies include medical histories in their models, they only use high-level information from previous encounters (eg, the number of inpatient stays in the previous year) instead of detailed information such as previous laboratory test results. By using the patients’ detailed medical history, we were able to add more variables to the model without sacrificing its timeliness. As a result, our model enables point-of-care prediction and can be used to continuously monitor the readmission risk during the entire episode of hospitalization.

### Generalizability

In addition to the performance of the model, we considered its generalizability. From the modeling point of view, generalizability indicates if a model can achieve similar performance on new data. In other words, the model should be trained and built using a large and diverse training sample to represent the whole population. Most existing readmission prediction models were based on relatively homogenous (eg, single-center studies) and small (eg, less than 20,000 patients) samples. For example, the LACE index and the HOSPITAL score were derived from only 4812 Canadian and 9212 American patients, respectively [13,14]. To ensure good generalizability, we captured all eligible inpatient encounters in 2016 from the Health Facts database, with 96,550 patients discharged from 205 hospitals across the four US Census regions. The best performing model (XGBoost) has a validation AUC close to the mean 10-fold cross-validation AUC on the development set (0.742 vs 0.753). This indicates that the model has good generalizability.

Another consideration of generalizability is whether the model can work on various types of patients. There is no consensus on data inclusion criteria for readmission studies, and the study outcomes span condition- or procedure-specific to all-cause readmission predictive models [27]. The choice between these two types of models has long been under debate. In two systematic reviews [8,12] of 99 readmission predictive models reported between 1985 and 2015, 77% of the models were specialized for one patient subpopulation. The condition-specific design limits the adaptability of the models to other patient subpopulations and may overlook patients in some at-risk minority groups if specific models are not available [49,50]. In practice, it can be challenging for a hospital to maintain separate readmission prediction models for different patient subpopulations, and this situation will be further exacerbated if patients have comorbidities [50]. All-cause models are designed for broad patient populations without limiting diagnoses or procedures. In this work, we were interested in hospital-wide readmissions caused by medical and health care–related reasons. Our model is not specific to any conditions or procedures because we wanted to use it as an early screening tool to assess all patients' risk.

### Limitations

Although our model was designed to be nonspecific to patient populations, it does not work for patients under 18 years of age. This is because infant and pediatric readmissions were reported to have different patterns from adult readmissions [12,51] and could be influenced by parental factors [51,52]. The Health Facts database is deidentified, and there is no information about the patients’ families. Therefore, we removed patients aged younger than 18 years from the data. In addition, the Health Facts database only contains data collected from US health care settings. For readmissions in other countries, where patient demographics (eg, race) and medical interventions (eg, medications) are different from those in the United States, our model may not work well.

Another limitation was that the 14 novel risk factors and 2 protective factors were identified based on associations. Because this work was a retrospective study on deidentified data, we were not able to further investigate the relationship between our findings and factors reported in other studies.

### Conclusions

In this work, we developed an early prediction model for unplanned 30-day hospital readmission. The model has better performance (AUC of 0.753 on the development data set and 0.742 on the validation data set) and timeliness than established readmission models such as the HOSPITAL score, LACE index, and LACE-rt index. The model was derived and validated from a large and diverse patient population (96,550 patients discharged from 205 hospitals across four US census regions), and it can be generalized in use for adult patients in the United States. We identified 14 novel risk factors and 2 novel protective factors of readmission that may shed light on the understanding of the complex readmission problem. More studies or trials are necessary to verify the relationship of these factors with readmission in the future.

## Appendix

Multimedia Appendix 1 Features of the XGBoost model.

Multimedia Appendix 2 Multivariate logistic regression analysis.

## Abbreviations

ADTree: alternating decision tree
AUC: area under the receiver operating characteristic curve
CCS: Clinical Classifications Software
CMS: Centers for Medicare & Medicaid Services
COPD: chronic obstructive pulmonary disease
CPT: current procedural terminology
EHR: electronic health record
HCPCS: Healthcare Common Procedure Coding System
HIPAA: Health Insurance Portability and Accountability Act
HRRP: hospital readmissions reduction program
ICD-10-CM: International Classification of Diseases, Tenth Revision, Clinical Modification
ICD-10-PCS: International Classification of Diseases, Tenth Revision, Procedure Coding System
XGBoost: extreme gradient boosting
