# Supplementary material for: Early Prediction of Unplanned 30-Day Hospital Readmission: Model Development and Retrospective Data Analysis
Source: JMIR Med Inform. 2021 Mar 23;9(3):e16306. doi: 10.2196/16306 (PMC8077543; doi:10.2196/16306)

Multimedia Appendix 1. Features of the XGBoost model.

(1) Demographics and index admission factors.

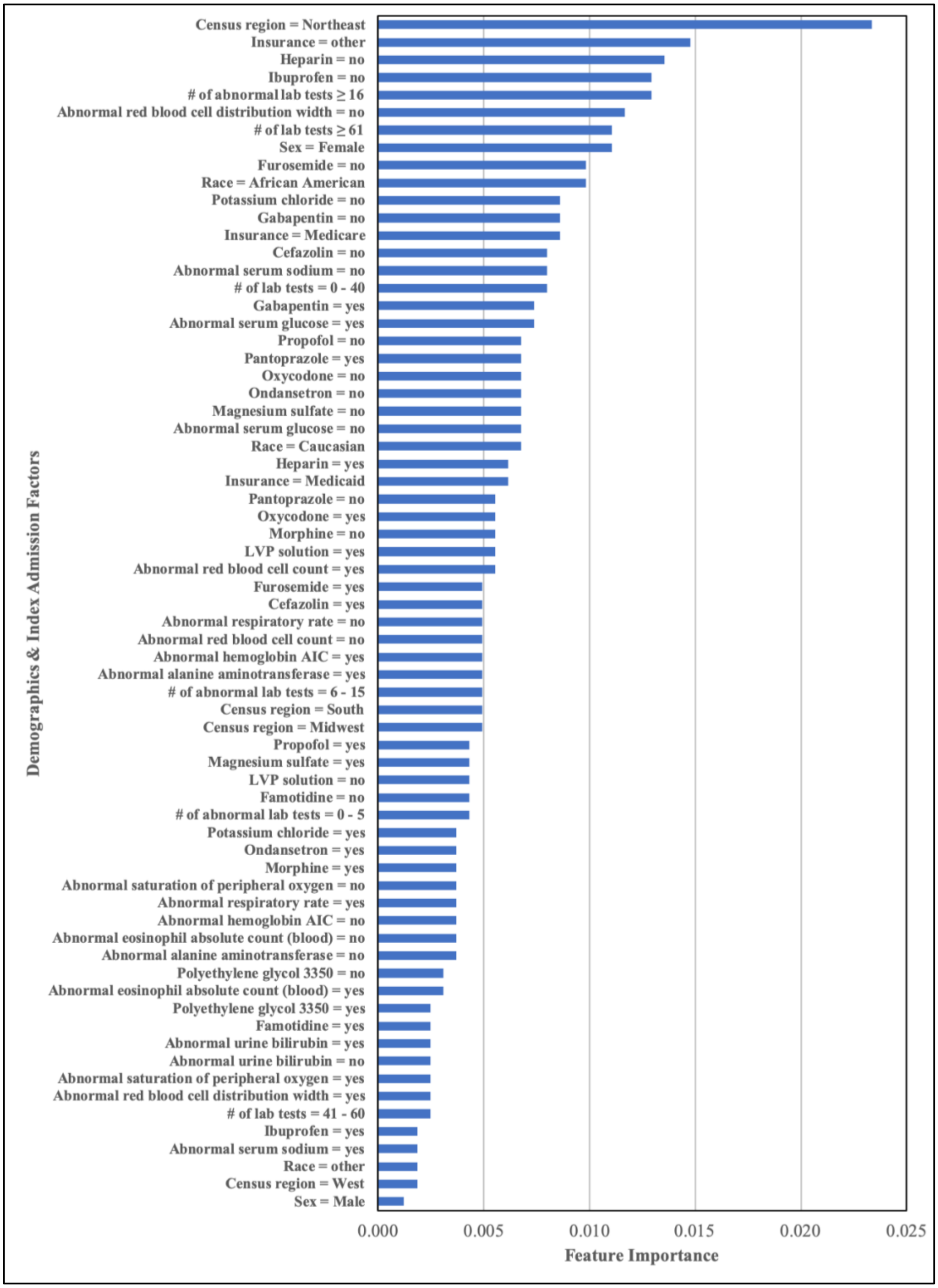

(2) Medical history (last 12 months) factors (Part 1).

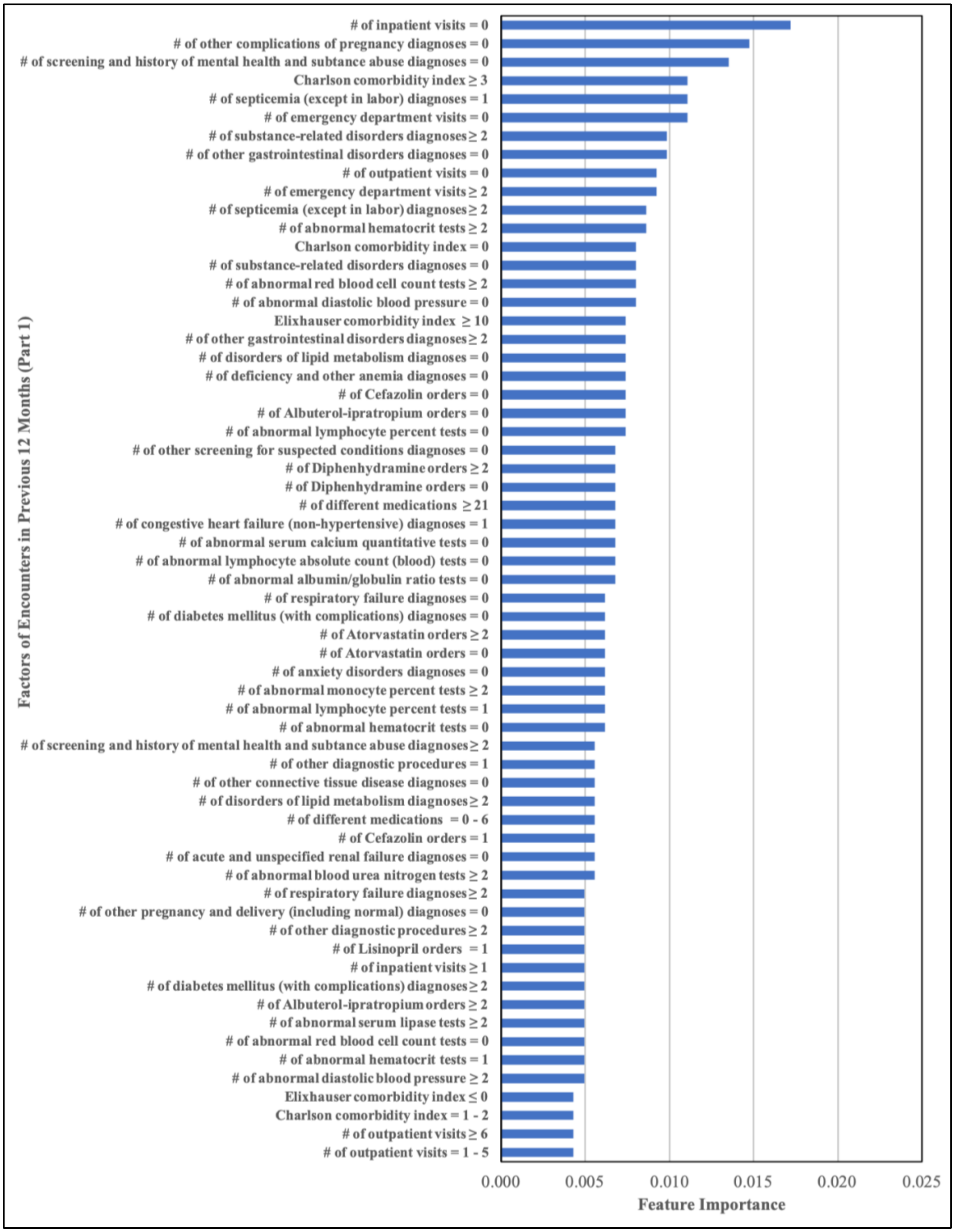

(3) Medical history (last 12 months) factors (Part 2).

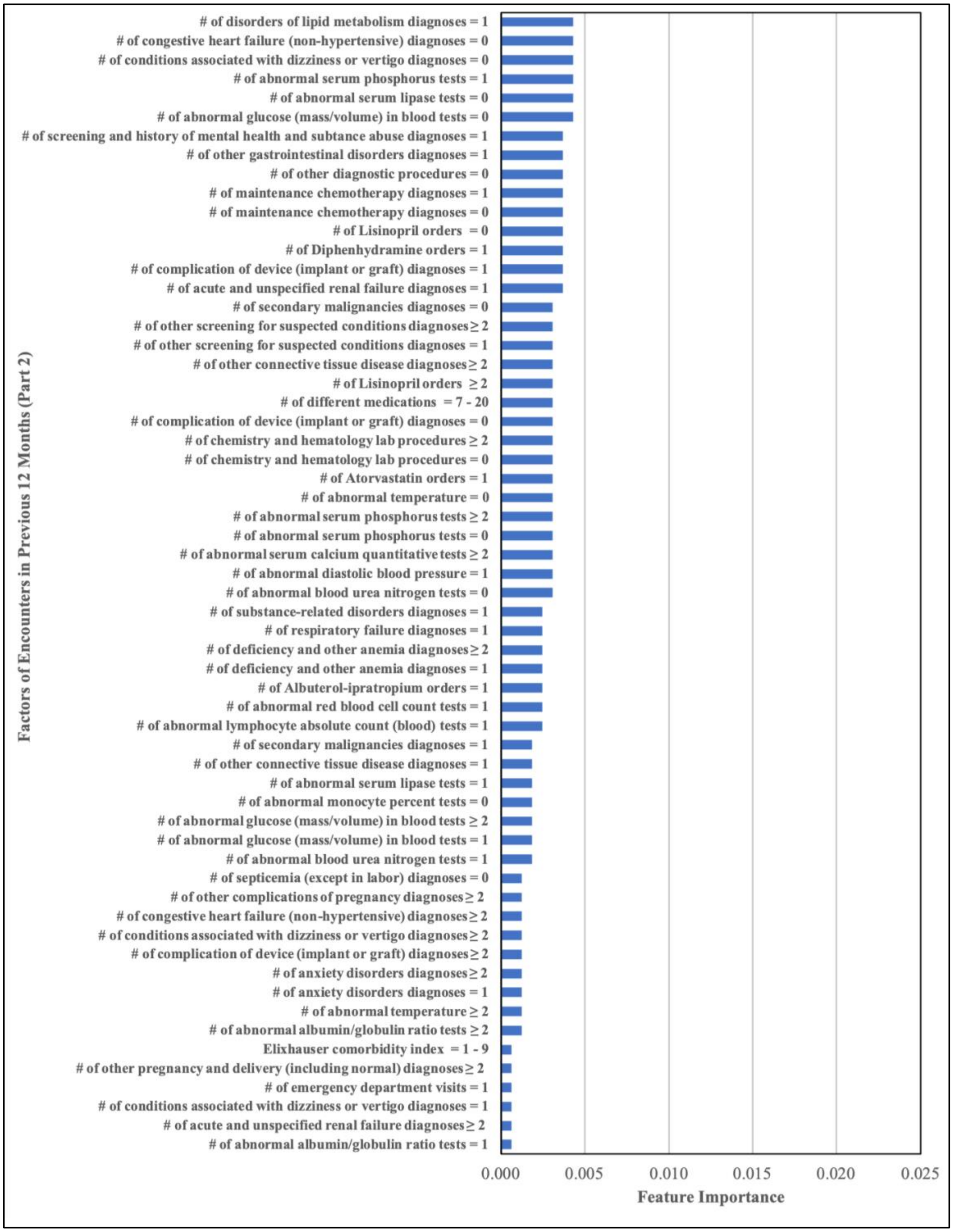

Supplement: Multimedia Appendix 1 [file medinform_v9i3e16306_app1.pdf]
